# Absence of detrusor muscle in TUR-BT specimen – can we predict who is at highest risk?

**DOI:** 10.1186/s12894-023-01278-7

**Published:** 2023-06-07

**Authors:** Yannic Volz, Rabea Trappmann, Benedikt Ebner, Lennert Eismann, Nikolaos Pyrgidis, Paulo Pfitzinger, Robert Bischoff, Boris Schlenker, Christian Stief, Gerald Bastian Schulz

**Affiliations:** Department of Urology, Ludwig-Maximilian-University Hospital , Munich, Germany

**Keywords:** Urinary bladder neoplasms, Carcinoma, Transitional cell, Quality control, Disease-free survival, Pathology, Surgical

## Abstract

**Introduction:**

As a high-quality TUR-BT is important to ensure adequate treatment for bladder cancer patients, the aim of the current study is to investigate the impact of patient-related, surgical and tumor-specific parameters on detrusor muscle (DM) absence (primary objective) and to assess the impact of DM on the prognosis after a TUR-BT (secondary objective).

**Patients and methods:**

Transurethral resection of bladder tumors (TUR-BTs) between 2009 and 2021 were retrospectively screened (*n* = 3237). We included 2058 cases (1472 patients) for the primary and 472 patients for secondary objective. Clinicopathological variables including tumor size, localization, multifocality, configuration, operation time and skill-level of the urologist were assessed. We analyzed predictors for missing DM and prognostic factors for recurrence-free survival (RFS) for the complete cohort and subgroups.

**Results:**

DM was present in 67.6% (*n* = 1371/2058). Surgery duration (continuous, minutes) was an independent predictor for absence of DM in the complete cohort (OR:0.98, r:0.012, 95%CI:0.98–0.99, *p* = 0.001). Other significant risk factors for missing DM were papillary tumors (OR:1.99, r:0.251, 95%CI:1.22–3.27, *p* = 0.006) in the complete cohort and bladder-roof and posterior-bladder-wall localization for re-resections. Absence of DM in high-grade BC correlated with reduced RFS (HR:1.96, 95%CI:1.0–3.79, *p* = 0.045).

**Conclusion:**

Sufficient time for a TUR-BT is mandatory to assure DM in the TUR-BT specimen. Also, cases with more difficult locations of bladder tumors should be performed with utmost surgical diligence and endourological training should incorporate how to perform such operations. Of note, DM correlates with improved oncological prognosis in high-grade BC.

**Supplementary Information:**

The online version contains supplementary material available at 10.1186/s12894-023-01278-7.

## Introduction

Three out of four patients present with non-muscle invasive bladder cancer (NMIBC) at first diagnosis [1]. At this stage, long-term curative treatment can be achieved in the majority of cases. A high-quality transurethral resection of the bladder tumor (TUR-BT) assures accurate local staging and is a keystone for the allocation of further treatment algorithms [2–4].

The skills level of the endourological surgeon [5, 6], completeness of the TUR-BT [7], but especially capturing of vesical detrusor muscle (DM) [3, 5, 8, 9] were correlated with better prognosis. Furthermore, the usage of liquid biopsies or biochemical markers have been shown to impact diagnosis and prognosis of bladder cancer patients [10, 11]. An evidence-based consensus statement lists the inclusion of DM in the pathological specimen as a quality indicator for a TUR-BT, as missing DM implies the risk of understaging as well as residual disease [12].

Further evidence underlines the significance of DM. In a study including pT1G3 high-grade BC absence of DM was an independent predictor for progression to muscle-invasive BC [9]. Gontero et al. proposed the omission of a re-resection in pT1 BC, if DM is present in the primary TUR-BT [13]. In another study including 365 patients Mariappan demonstrated lower recurrence-free survival (RFS) rates for patients with missing DM [8]. Of note, the current EAU (European Association of Urology) guideline on NMIBC only recommends a re-resection in pTa high-grade bladder cancer (BC) if DM is absent [1]. Several investigations reported significantly higher rates of residual disease in re-resections in patients with DM present vs. absent [5, 8, 14–16]. The risk for early recurrence or residual disease may be as high as two-fold, when DM was absent at the initial resection [8]. Even in small papillary tumors the risk of residual disease has been associated with absent DM [5].

So far different approaches to assure DM inclusion and improve the quality of the TUR-BT have been studied. These include the adherence to predefined quality indicators with improved oncological outcome [14], the implementation of surgical scorecards, where urologists could see their individual performance, raising awareness about DM presence, [17] or perioperative checklists [15], en-bloc resection and virtual reality endourological training [18, 19].

Although these approaches are important, it seems to be clinically relevant to identify specific constellations, where the risk of missing DM is higher. Training programs, allocation of operations to different skill levels, but also individual urologists’ awareness could profit from this information. To date, only few studies with a limited number of patients correlated clinical parameters with the absence of DM [3, 20]. The current study therefore aims to conduct a large cohort study investigating predictors for missing DM. Furthermore, we assessed the prognostic impact of absence of DM in different risk-groups.

### Patients and methods

The current study is based on a retrospective dataset of consecutive TUR-BT between 03/2009 and 02/2021 at our tertiary referral center. Data included baseline demographic, clinical and pathological variables. Clinical and surgery-specific variables such as “tumor configuration”, “tumor size” or “location” were obtained from the respective surgical report. Demographic variables such as “age”, “sex”, “comorbidities” or “previous TUR-BTs” were collected from the medical record.

Exclusion criteria from the final analysis were the absence of a macroscopic tumor during the cystoscopy at TUR-BT, suspicion for carcinoma-*in-situ* only as well as missing information about the absence/presence of DM in the histopathological report.

Oncological follow-up was performed by either re-TUR-BTs or cystoscopies at our institution.

Pathological assessment was performed according to the WHO classification from 2004/2016 and 1973. Information regarding the inclusion of DM was retrieved from the histopathological report.

The primary objective of the study is to investigate predictive factors for the presence of DM. We included tumor specific variables defining the complexity of the operation including tumor size (< / ≥ 3 cm), tumor location (bladder-wall, bladder-roof, posterior bladder-wall and proximity to ureteral orifice), the number of previous TUR-BTs, tumor configuration (papillary/flat vs. solid), multiplicity of tumors and the presence bladder diverticula/trabecula. Furthermore, we evaluated parameters which can be influenced including the use of relaxation, the experience of the surgeon (resident in training vs. board-certified urologist), the duration of the surgery as well as the timing of surgery (morning vs. afternoon). At last, we included patient-related variables (age, BMI and gender). For the primary objective, subgroup analyses were performed for the following groups: all patients, primary diagnosis only, high-grade urothelial cell carcinoma only, patients with NMIBC only, re-resection within 6 weeks, and patients with stroma-invasive tumors (pT1) only.

The secondary objective was to define prognostic parameters (including the presence of DM) for the oncological outcome were assessed. Only patients with the primary diagnosis of BC were included in this analysis. Potential prognostic parameters included were sex, smoking history, tumor multiplicity, tumor size, tumor configuration, the use of PDD (photodynamic diagnostis), the presence of bladder diverticula/trabecula, stroma-invasive tumor stage (pT1) and tumor location (bladder wall, bladder roof, posterior bladder wall and proximity to ureteral orifice). Recurrence-free survival was employed as endpoint and was defined as histopathological evidence of a low-grade and/or high-grade BC. For the secondary objective, we analyzed all patients, patients with NMIBC only, patients with stroma-invasive tumors (pT1) only and patients with high-grade tumors only.

Correlation of clinicopathological variables and DM status were conducted with the chi-square test and Mann–Whitney-U test. Only in the case of significant results in univariate analysis, backward selection multivariate analysis was performed by binary logistic regression. Survival was compared by using Kaplan–Meier-curves and log-rank test. Normality was assessed using the Kolmogorov–Smirnov test. Predictors for RFS were identified using Cox-logistic regression analysis, if univariate tests were statistically significant. A *p*-value ≤ 0.05 was considered significant and reported two-sided. All statistical analyses were performed using SPSS (Version 26.0, IBM). Ethical approval was obtained by the local ethical committee board (No.: 20–1090).

## Results

### Cohort characteristics

**Fig. 1:**
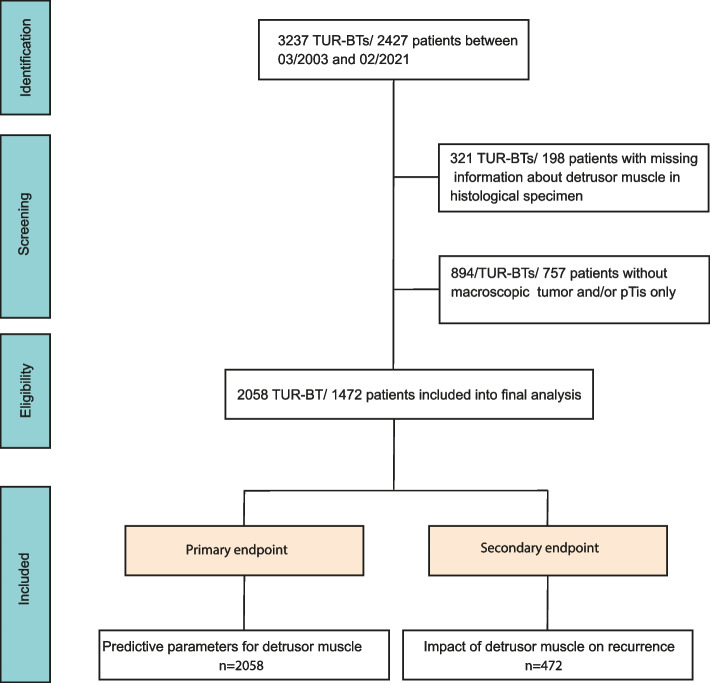
Flow-chart of the patient selection process

**Fig. 2:**
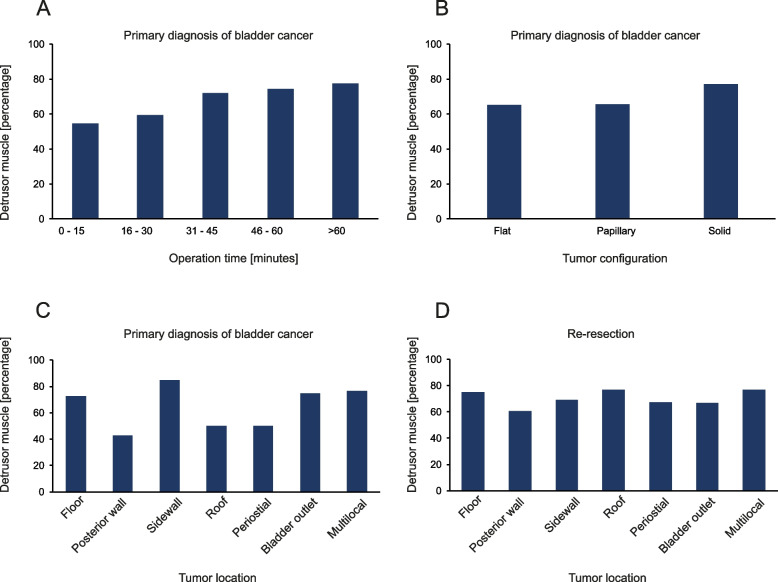
Prevalence of detrusor muscle in different clinical situations. Proportion of detrusor muscle present in the histological specimens in relation to: duration of surgery at primary diagnosis **A**, tumor configuration at primary diagnosis **B**, location of the tumor at primary diagnosis **C** and location of the tumor in re-resections **D**

Screening identified 3273 TUR-BTs. Overall, we included 2058 TUR-BTs/1472 patients (primary objective) and 472 patients (secondary objective) into the final analyses (Fig. 1). 198 patients were excluded due to missing information about DM and 757 patients were excluded due to no visible tumor or carcinoma-in-situ suspicion only. DM was present in 67.6% (1391/2058) and absent in 32.4% cases (667/2058). The median age of the total cohort was 72 years (IQR: 63–79) and 78.7% (*n* = 1158/1472) and 21.3% (314/1472) were male and female, respectively. Median follow-up time was 56 weeks (IQR 11–149 weeks). TUR-BTs were performed by 64 urologists. Patients who underwent obturatorius relaxation during their surgery were more likely to have DM present (73.4% vs. 62.7%, *p* < 0.001). TUR-BTs with DM lasted significantly longer (44 vs. 36 min, *p* < 0.001). Regarding tumor specific variables, multiple vs. unifocal tumors (70.7% vs 65.0%; *p* = 0.006) and high-grade vs. low-grade tumors (75.2% vs. 65.9%; *p* < 0.001) had a higher likelihood of DM in the histological specimen. Also, we have found DM more frequently in larger tumors and with increasing tumor depth. Interestingly, there was no difference between junior and senior surgeons regarding the presence of DM (44.8% vs. 47.5%, *p* = 0.258). In 63 TUR-BTs there was no malignancy (pT0) in the histological specimen. However, these were all re-resections. Regarding previous intravesical therapies we did not find any significant difference in the presence of DM for BCG (*p* = 0.054) and Mitomycin C (*p* = 0.726). Further key patient characteristics are displayed in Table 1 and Fig. 2.

**Table 1:** Association of missing detrusor muscle with clinicopathological parameters. TUR-BT – Transurethral resection of the bladder tumor, BMI – Body Mass Index, ASA—American Society for Anesthesiologists

	**Total**	**No detrusor muscle in specimen**	**Detrusor muscle in specimen**	***p*-value**
No. of TUR-BTs	2058 (100.0)	667 (32.4)	1391 (67.6)	
Age [yrs; median (IQR)]	72 (63 – 79)	71 (61—71)	72 (64 – 79)	**0.011**
Sex [n (%)]				
Male	1619 (78.7)	518 (32.0)	1101 (68.0)	0.440
Female	439 (21.3)	149 (33.9)	290 (66.1)	
BMI [median (IQR)]	26 (23.3 – 29.0)	26 (23.0 – 28.7)	26 (23.6 – 29.0)	0.651
Smoking history [n (%)]	829 (54.9)	271 (32.8)	558 (67.2)	0.897
Oral anticoagulation [n (%)]	745 (43.5)	223 (29.9)	522 (70.1)	0.183
Anesthesia [n (%)]				
General	1463 (94.6)	449 (30.7)	1014 (69.3)	0.987
Regional/Spinal	80 (5.2)	23 (30.0)	56 (70.0)	
Other	3 (0.2)	1 (33.3)	2 (66.7)	
ASA score [n (%)]				
≤ ASA 2	684 (38.4)	235 (34.4)	449 (65.6)	**0.045**
≥ ASA 3	1097 (61.6)	327 (29.8)	770 (70.2)	
Relaxation [n (%)]				
No	598 (38.8)	223 (37.3)	375 (62.7)	** < 0.001**
Yes	945 (61.2)	251 (26.6)	694 (73.4)	
Photodynamic diagnostic [n (%)]	1508 (73.3)	491 (32.6)	1017 (67.4)	0.882
Duration of surgery [min; median (IQR)]	41 (28 – 59)	36 (25 – 54)	44 (30 – 60)	** < 0.001**
Timing of surgery [n (%)]				
Morning (08–12)	1122 (60.3)	345 (30.7)	777 (69.3)	0.266
Afternoon (12–16)	739 (39.7)	246 (33.2)	495 (66.8)	
Tumor size [n (%)]				
< 1 cm	493 (33.2)	201 (40.8)	292 (59.3)	** < 0.001**
1 to 3 cm	363 (24.4)	111 (30.6)	252 (69.4)	
> 3 cm	630 (42.4)	153 (24.3)	477 (75.7)	
< 3 cm	856 (57.6)	312 (36.4)	544 (63.6)	** < 0.001**
≥ 3 cm	630 (42.4)	153 (24.3)	477 (75.7)	
Tumor multiplicity [n (%)]				
Single	1133 (55.1)	396 (35.0)	737 (65.0)	**0.006**
Multiple	925 (44.9)	271 (29.3)	654 (70.7)	
Tumor stage [n (%)]				
pT0	63 (4.2)	23 (36.5)	40 (63.5)	** < 0.001**
pTa	781 (51.9)	258 (60.4)	523 (48.5)	
pT1	400 (26.6)	133 (33.3)	267 (66.8)	
pT2	241 (16.0)	2 (0.8)	239 (99.2)	
pT3	0 (0.0)	0 (0.0)	0 (0.0)	
pT4	21 (1.4)	11 (52.4)	10 (47.6)	
Grading [n (%)]				
Low-grade	621 (41.1)	212 (34.1)	409 (65.9)	** < 0.001**
High-grade	891 (59.9)	221 (24.8)	670 (75.2)	
Histology [n (%)]				
Urothelial	1473 (71.6)	423 (28.7)	1050 (71.3)	** < 0.001**
Squamous cell	26 (1.3)	10 (38.5)	16 (61.5)	
Adeno-Ca	6 (1.3)	1 (16.7)	5 (83.3)	
Other	11 (0.5)	2 (18.2)	9 (81.8)	
No malignancy	542 (26.3)	231 (42.6)	311 (57.4)	
Concomitant Carcinoma in situ [n (%)]	281 (18.6)	73 (26.0)	208 (74.0)	0.441
Surgeon category				
Senior	1380 (67.5)	725 (52.5)	655 (47.5)	0.258
Junior	663 (32.5)	366 (55.2)	297 (44.8)

### Predictors for the presence of detrusor muscle in the histopathological specimen

The primary endpoint of the study was to establish predictive factors for the absence/presence of DM. Several patient- and tumor-specific parameters, which either characterize the degree of difficulty of the TUR-BT or can be influenced by the urologist were included. Multivariate analysis for the total cohort revealed age (OR 0.98; 95%CI 0.97–0.99; *p* = 0.014, B -0,017), surgery duration (minutes, continuous; OR 0.99; 95%CI 0.98–0.99; *p* = 0.001, B -0.012) and papillary vs. solid tumor configuration (OR 1.99; 95%CI 1.22–3.27; *p* = 0.006) as independent predictors for the absence of DM (Table 2). Similar results were found in patients with first diagnosis only (duration: OR 0.98; 95%CI 0.97–0.99; *p* = 0.002, B -0.016; configuration: OR 1.91; 95%CI 1.02–3.61; *p* = 0.043). In line with the previous results, we found increasing duration of surgery (OR 0.99; 95%CI 0.97–0.99; *p* = 0.002, B -0.012) and tumor configuration (OR 2.23, 95%CI 1.18–4.20, *p* = 0.013) to be predictors for finding DM in high-grade urothelial cell carcinoma only. For re-resections we found location (bladder-roof) and number of previous TUR-BTs to influence the absence of DM independently (OR 4.60; 95%CI 1.50–24.70; *p* = 0.012 and OR 4.99; 95%CI 1.28–19.44); *p* = 0.021, respectively). In the subgroups NMIBC tumors only and stroma-invasive (pT1) tumors only we did not find independent predictors for DM.

**Table 2:** Multivariate analysis of predictors for the absence of DM in the histological specimen in the total cohort and subgroups

**Complete cohort**				
Variable	Hazard ratio (HR)	R	Confidence interval 95% (CI95%)	*P*-value
Age	0.98	-0.016	0.97 – 0.99	**0.014**
Duration of surgery (minutes)	0.99	-0.012	0.98 – 0.99	**0.001**
Configuration (papillary vs. Solid)	1.99	0.691	1.22 – 3.27	**0.006**
**Primary diagnosis**				
Variable	Hazard ratio (HR)	R	Confidence interval 95% (CI95%)	*p*-value
Duration of surgery (minutes)	0.98	-0.016	0.97 – 0.99	**0.002**
Configuration (papillary vs. solid)	1.91	0.651	1.02 – 3.61	**0.043**
**High grade bladder cancer**				
Variable	Hazard ratio (HR)	R	Confidence interval 95% (CI95%)	p-value
Duration of surgery (minutes)	0.99	-0.012	0.97 – 0.99	**0.021**
Configuration (papillary vs. Solid)	2.23	0.801	1.18 – 4.20	**0.013**
**NMIBC**				
Variable	Hazard ratio (HR)	*R*	Confidence interval 95% (CI95%)	*p*-value
Duration of surgery (minutes)	0.99	-0.006	0.98 – 1.03	0.192
**Re-resection**				
Variable	Hazard ratio (HR)	R	Confidence interval 95% (CI95%)	*p*-value
Location (posterior bladder wall)	4.60	1.525	0.96 – 22.05	0.057
Location (bladder roof)	6.06	1.801	1.50 – 24.70	**0.012**
No. of TUR-BT	4.99	1.607	1.28 – 19.44	**0.021**
**Stroma-invasive (pT1) tumors**				
Variable	Hazard ratio (HR)	R	Confidence interval 95% (CI95%)	*p*-value
Duration of surgery (minutes)	0.99	-0.013	0.97 – 1.02	0.096

### The presence of detrusor muscle and its impact on recurrence-free survival

We next investigated, if the presence of DM (Supp. Figure 1) next to other variables has a major and independent impact on the oncological prognosis in the different clinical subgroups.

**Fig. 3:**
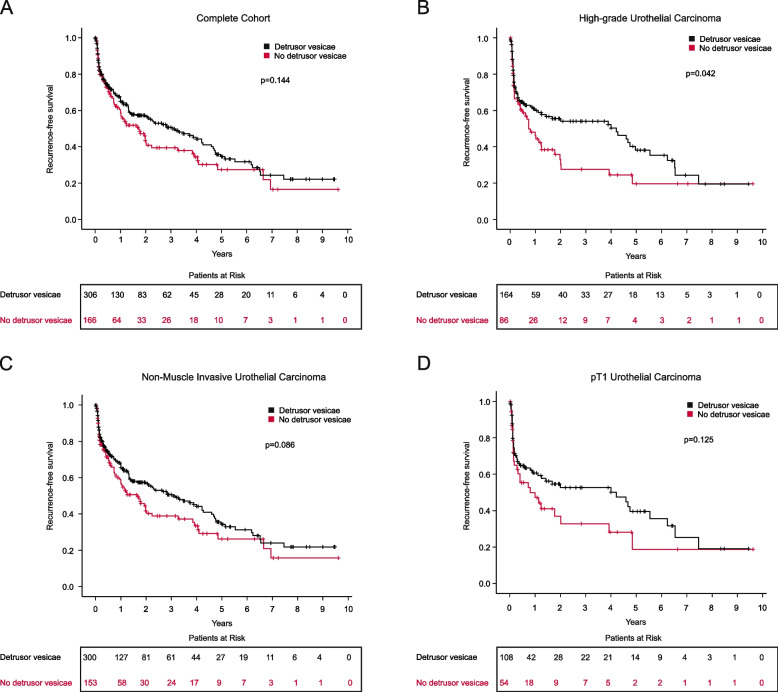
Impact of detrusor muscle on the oncological prognosis. Survival curves and patients at risk in case of the absence/presence of DM in: the total cohort **A**, high-grade urothelial cell carcinoma only **B**, NMIBC only **C** and stroma-invasive tumors (pT1) only **D**

For the subgroup of high-grade tumors, RFS was significantly longer when DM was present (*p* = 0.042). Median RFS for patients with DM was 219 weeks and for patients without DM 38 weeks. The presence of DM did not lead to a difference in RFS in the total cohort, in the non-muscle invasive BC group and the subcohort of stroma-invasive BC (Fig. 3). In the multivariate analysis for RFS absence of DM was an independent prognostic factor in patients with high-grade tumors (HR 1.96; 95%CI 1.02–3.79; *p* = 0.045). Other variables such as sex, tumor location and bladder diverticula did not impact RFS (Table 3). In sum, the absence of DM significantly decreases RFS in a very delicate group of patients.

**Table 3:** Multivariate analysis for prognostic factors of RFS

Variable	Hazard ratio (CI95%)	R	*p*-value
Sex	3.25 (0.76 – 13.92)	1.180	0.112
Detrusor muscle missing	1.96 (1.02 – 3.79)	0.673	**0.045**
Location (posterior bladder wall)	0.48 (0.20 – 1.12)	-0.742	0.088
Bladder diverticula	2.77 (0.97 – 7.91)	1.020	0.056

## Discussion

A TUR-BT of high-quality is of utmost importance as it provides accurate staging for patients with BC and therefore is the basis of further treatment decisions [2, 21]. Additionally, a broad body of evidence shows complete tumor resection including DM to be vital for the oncological prognosis [3, 7, 8, 14, 17, 22].

### Predictive markers for presence of detrusor muscle in TUR-BT specimen

In the current cohort DM was found in 67.7% of the cases, which corresponds with similar studies, reporting a DM positivity rate of 64% [15], 69.1% [23], 74.2% [17], and 90.4% [22]. In the univariate analysis, the likelihood of DM was significantly higher for larger tumors, high-grade tumors and multiple tumors. Presence of DM also increased with invasion depth. The current results confirm the findings made by previous groups [3, 8, 9, 14]. Shoshany et al*.* included 332 patients undergoing TUR-BT and found larger tumor size, multifocal BC, higher tumor grade, and solid tumors associated with a higher rate of DM [3]. In a cohort of 356 patients Mariappan et al*.* [8] demonstrated tumor size and high-grade tumors to be predictive for DM. In a larger cohort, the same group reported increased hospital case volume next to multiple tumors correlated with DM [14].

Interestingly, several studies showed that urologists have a good accuracy in predicting stage and grade of BC during cystoscopy [24, 25]. Therefore, urologists might be more ambitious to resect DM if they suspect invasion of the lamina propria or muscle-invasive disease. On the other side, if a low-risk NMIBC is probable, the surgeon might weigh the prevention of a bladder perforation higher compared to capturing DM. Furthermore, in solid tumors the surgeons may act more confident, as a perforation is less likely. Interestingly, our study revealed no difference between “junior” and “senior” surgeons, which is on the contrary to results of Mariappan et al*.* [5, 8] and Roupret et al*.* [20]. On the other side, several studies did not show a significant impact of the skill level on the likelihood of finding DM [3, 22]. This seeming discrepancy might be due to different endourological training programs and supervision standards. Importantly, patients with DM present underwent intraoperative relaxation more frequently. This is of clinical interest, as urologists might be more confident to perform deep resections in vulnerable regions like the lateral bladder walls. In multivariate analyses, the number of previous TUR-BTs and tumor location at the bladder roof were independent predictors for an inferior rate of DM presence in patients undergoing re-resection. To our knowledge, this is the first time that a study analyzed the DM status in re-resections. Our finding can easily be explained by the higher risk of bladder perforations in these cases, and the consequences in the case of intraperitoneal perforation. Consequently, cases with such a high-risk situation should be preserved for urologists with excellent endourological skills. Of note, longer duration of the TUR-BT was independently associated with DM in the complete cohort, patients with first diagnosis and high-grade BC. To our knowledge, this is the first study investigating the impact of operation time on the quality of the TUR-BT and therefore is a key finding of our study. For patients undergoing radical cystectomy, the duration of time also seems to have an impact on outcome parameters [26]. However, it is evident that there is no linear relation between time and outcome. We propose that a TUR-BT needs sufficient time and should be performed with the same diligence as’major’ urological operation like cystectomy. In a time of increasing economic pressure on healthcare providers, our study provides important arguments against a’faster is better’ paradigm, as low-quality TUR-BT will lead to more recurrences leading to higher long-term costs. Taken together, tumor configuration and location, but also the diligence in the sense of time spent for the operation independently define the risk of DM absence in patients undergoing TUR-BT.

### Impact of detrusor muscle status on the oncological prognosis

The secondary objective of this study was the investigation of DM status on the prognosis. The current study was able to show that absence of DM was an independent prognosticator for reduced RFS in high-grade tumors. This association was not seen for the complete cohort, and the subgroups of NMIBC and pT1, although a non-significant trend was seen. Several other studies positively correlated DM in the TUR-BT specimen with improved RFS [3, 5, 8, 20], progression [9], and lower rates of residual disease [8, 14]. Of note, the other studies investigating recurrence included smaller cohorts and significantly less parameters. The rates of RFS in high-grade tumors in the current study seem long whenever DM is present. However, the results are well comparable to previous literature [3, 22, 27]. In line with our results, a retrospective analysis did not find a correlation between DM status and recurrence in low-grade pTa tumors [28]. In contrast to our study two other investigations demonstrated DM to be predictive of recurrence in patients with pT1 BC [3, 20]. This discrepancy might be explained by differences in the cohorts and follow-up time. Taken together, there is broad evidence that DM should be seen as a key quality indicator for high-grade BC.

To improve the quality of TURB-BTs it seems to be crucial to develop strategies that consistently lead to a higher frequency of DM present. Other approaches involving the introduction of scorecards/checklists also lead to a higher proportion of DM present and therefore a higher-quality TUR-BT [15–17]. The type of resection method used has also been shown to improve DM rates. Especially en-bloc resection and bipolar resections methods seem to yield better DM resection rates [18, 29].

The retrospective design is among the main limitations of this study and ideally demands prospective validation. Furthermore, inconsistencies of surveillance might mitigate the analyses regarding tumor recurrences. Also, we did not evaluate the occurrence of intraoperative/postoperative complications and therefore we cannot say whether the presence of DM might lead to a higher complication rate in specific tumor locations. Last, due to the relatively short follow-up time, we were not able to show a relevant number of progressions and therefore assess the impact of DM onto progression-free survival.

## Conclusion

Several risk factors for missing DM, including configuration, localization, and duration of operation, were identified. This might help urologists to better identify complex operations. Of note, this study provides robust evidence against an economic-driven ‘the faster, the better’ view on oncological operations. As the presence of DM influences the oncological outcome in high-risk tumors, TUR-BTs should be performed with the deserved diligence.

## Supplementary Information

**Additional file 1: Suppl. Figure 1. **Macroscopic appearance of detrusor vesicae muscle during TUR-BT. Histopathological examination revealed abundant detrusor muscle and a muscle-invasive urothelial carcinoma.

## Data Availability

The datasets generated and/or analysed during the current study are not publicly available due to ethical and legal obligations but are available from the corresponding author on reasonable request.
