# Supplementary figures and images for: Absence of detrusor muscle in TUR-BT specimen – can we predict who is at highest risk?
Source: BMC Urol. 2023 Jun 7;23:106. doi: 10.1186/s12894-023-01278-7 (PMC10249185; doi:10.1186/s12894-023-01278-7)

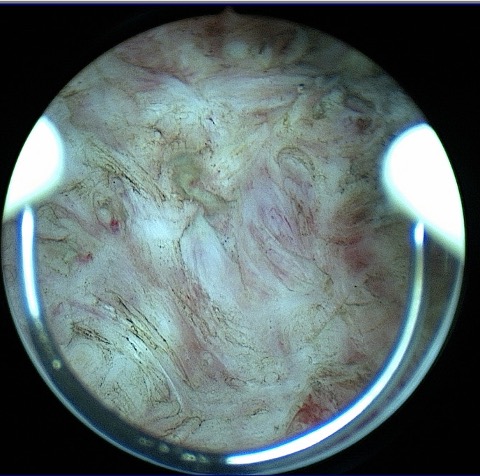

Supplement: Supplementary file 1 — Additional file 1: Suppl. Figure 1. Macroscopic appearance of detrusor vesicae muscle during TUR-BT. Histopathological examination revealed abundant detrusor muscle and a muscle-invasive urothelial carcinoma. [file 12894_2023_1278_MOESM1_ESM.jpg]
